# Exposure to Persistent Organic Pollutants Predicts Telomere Length in Older Age: Results from the Helsinki Birth Cohort Study

**DOI:** 10.14336/AD.2016.0209

**Published:** 2016-10-01

**Authors:** Maria Angela Guzzardi, Patricia Iozzo, Minna K. Salonen, Eero Kajantie, Riikka Airaksinen, Hannu Kiviranta, Panu Rantakokko, Johan Gunnar Eriksson

**Affiliations:** ^1^Institute of Clinical Physiology, National Research Council (CNR), Pisa, Italy; ^2^National Institute for Health and Welfare, Chronic Disease Prevention Unit, Helsinki, Finland; ^3^Children’s Hospital, Helsinki University Hospital and University of Helsinki, Helsinki, Finland; ^4^PEDEGO Research Group, MRC Oulu, Oulu University Hospital and University of Oulu, Oulu, Finland; ^5^Department of General Practice and Primary Health Care, University of Helsinki, Helsinki, Finland; ^6^Folkhälsan Research Centre, Helsinki, Helsingfors Universitet, Helsinki, Finland; ^7^Unit of General Practice, Helsinki University Hospital, Finland; ^8^National Institute for Health and Welfare, Department of Health Protection, Chemicals and Health Unit, Finland

**Keywords:** telomere length, pops, aging, environmental chemicals

## Abstract

As the population ages, the occurrence of chronic pathologies becomes more common. Leukocyte telomere shortening associates to ageing and age-related diseases. Recent studies suggest that environmental chemicals can affect telomere length. Persistent organic pollutants (POPs) are most relevant, since they are ingested with foods, and accumulate in the body for a long time. This longitudinal study was undertaken to test if circulating POPs predict telomere length and shortening in elderly people. We studied 1082 subjects belonging to the Helsinki Birth Cohort Study (born 1934-1944), undergoing two visits (2001-2004 and 2011-2014). POPs (oxychlordane, trans-nonachlor, p, p’-DDE, PCB 153, BDE 47, BDE 153) were analysed at baseline. Relative telomere length was measured twice, ’10 years apart, by quantitative real-time PCR. Oxychlordane, trans-nonachlor and PCB-153 levels were significant predictors of telomere length and shortening. In men, we did not find a linear relationship between POPs exposure and telomere shortening. In women, a significant reduction across quartiles categories of oxychlordane and trans-nonachlor exposure was observed. Baseline characteristics of subjects in the highest POPs categories included higher levels of C-reactive protein and fasting glucose, and lower body fat percentage. This is one of few studies combining POPs and telomere length. Our results indicate that exposure to oxychlordane, trans-nonachlor and PCB 153 predicts telomere attrition. This finding is important because concentrations of POPs observed here occur in contemporary younger people, and may contribute to an accelerated ageing.

The proportion of elderly individuals is increasing worldwide (Global brief for World Health Day 2012: Good health adds life to years (www.who.int/world_health_day/2012) [1]. Unfortunately, the increase in life-expectancy has not been accompanied by a parallel decline in the occurrence of chronic pathologies that in turn has become progressively more common (Global brief for World Health Day 2012: Good health adds life to years. www.who.int/world_health_day/2012).

Telomeres are DNA-protein complexes placed at the end of chromosomes, composed of tandem (5’-(TTAGGG) n-3’) repeats to protect chromosome stability and integrity, and avoid DNA damage. Telomere length progressively shortens within repeated cell divisions, and is therefore considered a major marker of cellular ageing [2]. In population studies, telomere length is commonly measured from peripheral blood leukocytes, and leukocyte telomere length (LTL) has been introduced as a marker of the ageing process in an individual. Notably, LTL associates to age-related diseases [3-9]. Therefore, telomere integrity contributes simultaneously to the preservation of health and the prolongation of life.

Recent studies support the concept that environmental chemicals can affect telomere length, and suggest that this mechanism may explain the link between pollution and chronic diseases [10]. Most of these studies have focused on occupational exposure to traffic-related (truck drivers) and industrial pollutants (rubber, lead, pesticide, petrol, and coke-oven workers) [10]. Thus, these findings pertain to very specific categories of individuals, whereas the relationship between telomere length and environmental chemicals in the general ageing population has not been addressed. In this context, lipophilic persistent organic pollutants (POPs) are most relevant due to their human exposure via food ingestion, their resistance against metabolism and following slow excretion [11-15]. These compounds accumulate in lipids of living organisms and become increasingly concentrated as they move up the food chain [14]. In the human body, POPs have a half-life of days to decades, and a majority of these toxicants accumulate along the life-course, contributing to the modulation of health through the ageing processes. Recent surveys (U.S. Environmental Protection Agency, Blood Persistent Organic Pollutants Level. http://cfpub.epa.gov/roe/indicator.cfm?i=65) underline that relevant concentrations of even legacy POPs still occur in young individuals [16-20], in spite of the fact that they were born after the release of these POPs in the environment had been restricted or banned [21, 22]. Because of their long persistence and unpredictable levels in foods [13], it is very difficult to estimate exposure levels to POPs in humans [20], and modulate intake by appropriate food or behavioural choices (WHO, Dioxins and their effects on human health. Fact sheet N°225, www.who.int/mediacentre/factsheets/fs225/en/).

The current longitudinal study was undertaken to test the hypothesis that circulating POPs predict telomere length and shortening in elderly people. This study was carried out in 1082 subjects belonging to the Helsinki Birth Cohort Study, which consists of men and women born between 1934 and 1944, in Helsinki, Finland, representing a general population of elderly people.

## MATERIALS AND METHODS

### Study population and design

**Figure 1.**
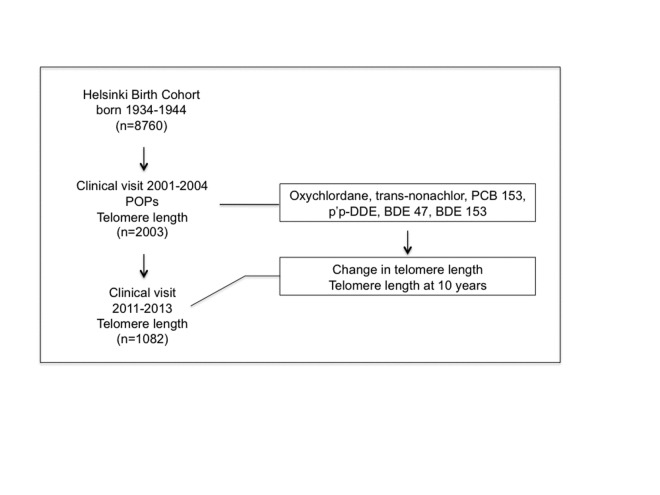
**Study design, showing the size of the population and the times of screening (left) and the predictive and outcome variables (right boxes).** We hypothesized that serum POPs levels would associate with telomere shortening, affecting telomere length in 10 years.

The original Helsinki Birth Cohort Study includes 8760 women and men born between 1934 and 1944 at the Helsinki University Hospital. Between 2001 and 2004, a randomly-selected subset of people from the cohort underwent a clinical visit (n=2003), during which blood samples were collected for the assessment of telomere length, circulating POPs and metabolic markers [23]. After approximately 10 years (2011-2013), a follow-up visit was carried out in 1082 subjects, and blood samples for DNA extraction and telomere length measurements were repeated (Fig. 1). Serum POPs levels were tested as predictors of telomere shortening during the observation period and of telomere length at 10 years (outcome measures). Age, sex, body fat percent and the inflammatory marker C-reactive protein (CRP) were tested as potential explanatory factors.

In the clinical study the subjects attended the clinic after an overnight fast. The clinical examination included measurements of height, weight, waist circumference, blood pressure, and body composition (Bioimpedance, InBody 3.0 eight-polar tactile electrode system, Biospace Co., Ltd, Seoul, Korea). Height and weight were measured in light indoor clothing and without shoes on. Height was measured to the nearest 0.1 cm and weight to the nearest 0.1 kg. Blood pressure was measured from the right arm while the subject was in the sitting position, and it was recorded as the mean of 2 successive readings from a mercury sphygmomanometer. Blood was drawn for measurements of lipids, glucose, inflammatory markers. A validated questionnaire was used to obtain information on medical history.

The studies were approved by the competent Ethics Committee at Hospital District of Helsinki and Uusimaa, and written informed consent was obtained from all subjects.

### DNA extraction and telomere length

Relative telomere length was measured twice, at the time of the first 2001-2004 visit, and after 10 years (2011-2014 follow-up visit) by quantitative Real-Time PCR. DNA was extracted from peripheral whole blood using a commercially available kit according to the manufacturer’s instruction (QIAamp blood Maxi kit and DNeasy blood and tissue kit, Qiagen s.r.l. respectively). DNA concentration and purity were assessed by calculating ultraviolet absorbance at 260/230 nm and 260/280 nm ratios. DNA integrity was tested by electrophoresis. No difference in DNA integrity and quality was reported between the two kits.

Relative leukocyte telomere length was determined by a quantitative real-time PCR-based method using a simple or a multiplex assay at the first and second time-point, respectively, as previously reported [24-26]. According to the simple real-time PCR assay method, quantification of telomere repeats copy number and of single copy gene copy number was performed in two separate runs as previously described [24, 27]. In the multiplex assay both copy numbers were assessed within the same plate and same run according to the method published by Cawthon et al. [25] with minimum modifications Briefly, DNA concentration was standardized to 4 ng/μl and PCR reactions were set-up in a 384-well plate (CFX384 Touch Real-Time PCR detection system, Bio-Rad Laboratories, CA, USA) and carried out in a final volume of 10 µl. Quality Control and threshold cycles (Ct) for both telomere and single copy gene (hemoglobin subunit beta) amplification were analyzed using a dedicated software (CFX Manager software, Bio-Rad Laboratories, CA, USA). All plates included three or four genomic DNA control samples used to calculate inter-assay coefficient of variation (CV), which was 21.0% at the first-time-point and 6.2% at the second time-point. Telomere length measured at the two time-points was significantly correlated to each other in the whole population (r=0.325, p<0.001) and in men (r=0.276, p<0.001) and in women (r=0.356, p<0.001) separately.

### Serum POPs and metabolic assays

The POPs analysed were oxychlordane, trans-nonachlor, 1,1-dichloro-bis (p-chlorophenyl)-ethylene (p,p’-DDE), 2,2’, 4,4’, 5,5’-hexachlorobiphenyl (PCB 153), and 2,2’, 4,4’-tetrabromodiphenyl ether (BDE 47), and 2,2’, 4,4’, 5,5’-hexabromodiphenyl ether (BDE 153). The analysis of POPs was carried out at the National Institute of Health and Welfare, Chemicals and Health Unit, which is accredited for the measurement of POPs in serum samples, according to the International Standard ISO/IEC 17025, as previously detailed [28]. All analyses were carried out in a blinded fashion. POP concentrations are given both in picograms per millilitre (wet-weight) and in picograms per milligram of circulating lipids (total cholesterol plus triglycerides). Serum cholesterol and triglyceride levels were measured by standard enzymatic methods, and plasma glucose was assayed by a hexokinase method. High-sensitivity CRP (hs-CRP) was analyzed with an immunoturbidimetric method, using Konelab T-serie High Sensitivity CRP analyzer (Thermo Fisher Scientific Oy, Vantaa, Finland).

### Statistical analysis

As shown in Fig. 1, we explored the hypothesis that serum POP levels would predict telomere shortening during the observation period and telomere length at 10 years (outcome). Data were examined in the whole cohort, and in women and men separately, by using SPSS for Mac OS X (version 20, Chicago, IL, USA). Non-parametric tests were mostly used and not normally distributed variables were log-transformed for parametric tests. Relative change in LTL over a 10-year time period was calculated adjusting for the baseline measurement (relative change percent in LTL = 100 * [LTL at 70 - LTL at 60]/LTL at 60). Data from POPs (2001-2004) and telomere length at the last visit were available in 1071 subjects, and data on 10 year changes in telomere length in 1047 subjects. POPs are mainly carried in the lipid component of the blood and lipid-standardized POP concentrations are frequently reported. However, POPs accumulation can disturb lipid metabolism itself and, as demonstrated in a simulation study [29], using wet-weight concentration and adjusting for total lipids would introduce less bias than using lipid-standardized concentrations [30, 31]. Serum total cholesterol and triglycerides were significantly correlated to serum wet-weight POPs levels (p<0.001) in the whole population and in the two genders separately. In the present study, both correlative and cross-sectional analyses were carried out using POPs wet-weight concentration (pg/ml), and triglycerides and total cholesterol were included as confounders [30, 31]. Analysis using lipid-standardized concentration led to same findings (not shown). A summary POPs variable was created from the three POPs species that resulted associated with telomere length (oxychlordane, trans-nonachlor, PCB153). Individual ranks of the three POPs were added and summary value was divided into four categories (quartiles). The Spearman’s correlation coefficient was used to determine associations between variables. Multivariate regression analyses were used to examine the confounding or explanatory effect of lipids, age or change in age between the two visits, and body fat percentage (at the baseline visit) in the association between telomere length and POPs. The confounding effect of presence of cardiometabolic disease (i.e. arterial hypertension, cardiac failure, coronary heart disease, type 2 diabetes) and cancer on the above association was also tested. The inflammatory marker CRP was examined as potential underlying factor for POPs-related telomere attrition. Comparisons between groups were performed by Mann-Whitney test. ANCOVA was used with normalized variables to adjust for the possible confounding effect of age, lipids and body fat percentage. Data are expressed as mean±SEM. A p value of less than 0.05 was considered statistically significant.

## RESULTS

### General characteristics in the whole cohort, and in men and women

The cohort (n=1082) was composed of 610 women (56%) and 472 men (44%). The characteristics of the cohort at the clinical visit in 2011-13 are given in Table 1. The age range was 67-79 years. In the whole population, the prevalence of hypertension, coronary heart disease, stroke, type 2 diabetes, and cancer was 31.5%, 6.7%, 1.1%, 5.5% and 4.9%, respectively at baseline. The corresponding numbers at follow-up were 49.1%, 9.1%, 2.8%, 15.0% and 15.2%, respectively. More detailed information on prevalence of chronic diseases has been reported in Guzzardi et al. [26]. Telomeres were on average 11% longer in women (p<0.001). Significant sex-related differences were observed in serum POPs concentrations, both when expressed as wet-weight and when standardized to lipid concentration. Specifically, oxychlordane, trans-nonachlor and PCB 153 concentrations were greater in men than women (p<0.001), p,p’-DDE and BDE 47 levels were similar between the sexes (p≥0.50), whereas BDE 153 level was greater in women. The association of POPs level and female gender was significant (p≤0.001) after adjusting for circulating lipids and for body fat percentage for all POPs categories with the exception of BDE 153 (p>0.05), consistent with a greater bioaccumulation of POPs in the more abundant adipose tissue observed in women.

### Relationship between POPs and telomere outcomes in the whole study cohort

As shown in Table 2, we explored the hypothesis that serum POPs levels would predict telomere shortening during the observation period and telomere length at 10 years (outcome). Matched data from POPs (2001-2004) and telomere length at the last visit were available in 1071 subjects (604 women, 56%, and 467 men, 44%), and 10 year changes in telomere length in 1047 subjects. Regression analyses were carried out to examine the exposure-response pattern of telomere length across POPs quartiles (trend) and across individual and summed values of three POPs that were associated with telomere length (oxychlordane, trans-nonachlor, PCB153) or shortening.

**Figure 2.**
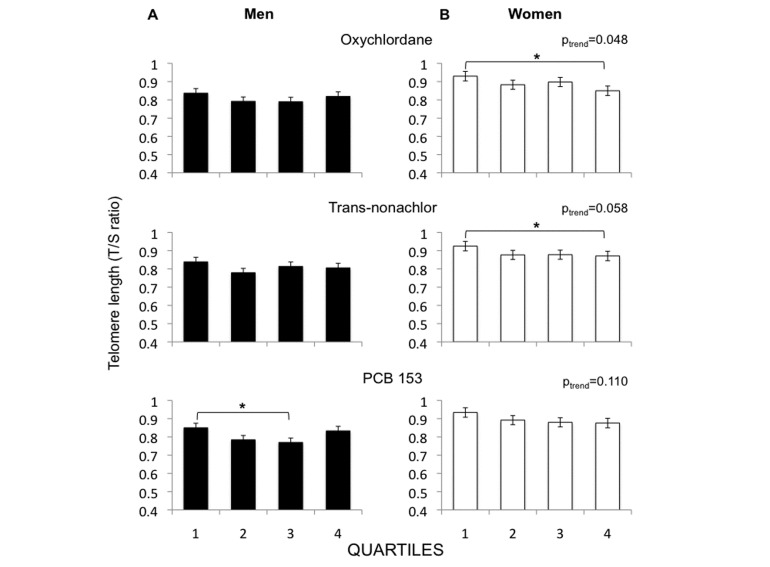
**Telomere length at the final visit, in relation to serum POPs concentrations as measured at baseline.** The population is stratified based on quartile categories of each POP in men (A) and women (B). Categories in men and women are calculated based on serum wet-weight POPs levels within each sex-group. Analysis is adjusted for total serum cholesterol and triglycerides levels. *p≤0.05.

In the whole study population, baseline (2001-2004) telomere length tended to be inversely correlated with quartile categories of oxychlordane (r=-0.056, p=0.070), PCB 153 (r=-0.060, p=0.051), p, p’-DDE (r=-0.056, p=0.071), and BDE-47 (r=-0.053, p=0.090) after correction for total lipids (Supplementary Table 1). Bivariate correlation between baseline telomere length and p’p’-DDE was statistically significant both when considering its serum concentration (p=0.048) and quartiles categories (p=0.033). However, adjustment for total lipids abolished the statistical significance. Correlations with other serum POPs concentrations did not reach statistical significance (p>0.15, Supplementary Table 1). In prospective data, oxychlordane, trans-nonachlor and PCB-153 levels were significant predictors of telomere shortening during the 10 years of observation (Table 2) and of short telomere length at 10 years (Table 2). Most of these findings remained significant after adjusting for total lipids, change in age between the two visits and/or body fat percentage at the baseline visit, and including the presence of cardio-metabolic diseases and cancer among the confounders (Table 2). Significance was similar if using absolute age instead of change in age (data not shown). As shown in Table 2 serum levels or quartile categories of p, p’-DDE, BDE 47 and BDE 153 were not significant predictors of telomere shortening or length at 10 years.

**Figure 3.**
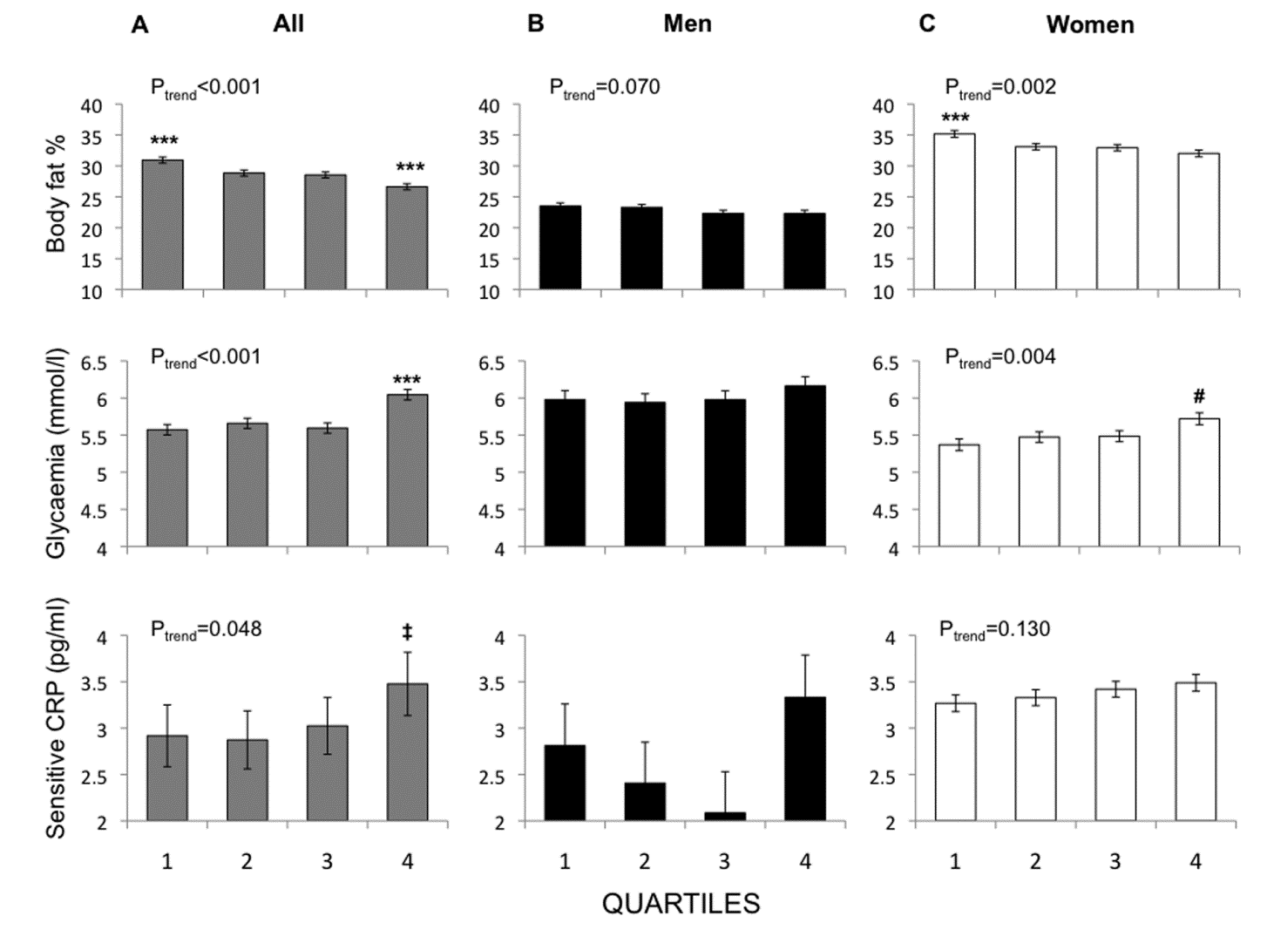
**Baseline measurements of body fat percentage (*top panels*), fasting plasma glucose (*middle panels*) and C-reactive protein levels (*bottom panels*), in relation to cumulative ranks of wet-weight serum POPs (oxychlordane, trans-nonachlor and PCB 153) concentrations as measured at the same baseline visit.** Comparisons are adjusted for total serum cholesterol and triglycerides, age (all) and fat % (glucose and CRP). Categories 1 to 4 correspond to quartiles. Categories in men and women are calculated based on serum POPs levels within each sex-group. ***p≤0.001, vs all other categories, ^‡^p=0.059, 0.042 vs categories 1 and 2, respectively, and ^#^p=0.005, 0.040, 0.069 vs category 1, 2 and 3 respectively.

After identifying the 3 POPs that were significant predictors of telomere attrition (oxychlordane, trans-nonachlor and PCB 153), their cumulative effect was examined by summing either their wet-weight concentrations or ranks and again stratifying the resulting sums into quartiles. As shown in Table 2, the cumulative POP concentration and score were significantly associated with a reduced telomere length at 10 years, independent of lipids, age, body fat percentage and diseases. Similarly, greater shortening during the 10-year observation period was significantly associated with cumulative POPs quartiles. Baseline (2001-2004) characteristics of subjects in the highest POPs categories included higher levels of C-reactive protein and fasting glucose, and lower body fat percentage (Fig. 3A).

**Table 1:** Clinical characteristics of the study cohort^°^

	All	Men	Women	p
Age (yrs)	71.0±0.1	70.8±0.1	71.1±0.1	0.218
Body mass index (kg/m2)	27.1±0.1	26.8±0.2	27.3±0.2	0.236
Height (cm)	168.3±0.3	176.2±0.3	162.2±0.2	<0.001
Waist-to-hip ratio	0.97±0.08	0.96±0.00	0.97±0.00	0.080
Lean body mass (kg)	53.5±10.6	63.2±0.4	46.1±0.2	<0.001
Body fat (%)	29.9±0.3	23.6±0.3	34.8±0.3	<0.001
Systolic BP	151.5±0.6	151.3±0.9	151.8±0.9	0.714
Diastolic BP	83.9±0.3	84.8±0.5	83.1±0.4	0.005
Mean BP	106.6±0.4	108.0±0.5	105.5±0.5	0.001
Total cholesterol (mmol/l)	5.39±0.03	5.10±0.05	5.62±0.04	<0.001
HDL (mmol/l)	1.62±0.01	1.46±0.01	1.74±0.01	<0.001
LDL (mmol/l)	3.21±0.03	3.06±0.04	3.32±0.03	<0.001
Triglyceride (mmol/l)	1.28±0.02	1.29±0.03	1.27±0.02	0.747
Telomere length (T/S)	0.855±0.009	0.807±0.012	0.893±0.013	<0.001
Oxychlordane (pg/ml)	43.8±0.6	48.3±1.0	40.4±0.8	<0.001
Trans-nonachlor (pg/ml)	114.7±2.0	136.1±3.3	98.1±2.2	<0.001
PCB 153 (pg/ml)	1103.6±15.1	1264.1±25.1	979.2±16.8	<0.001
p,p'-DDE (pg/ml)	2078.5±50.4	2072.7±66.8	2083.0±72.9	0.304
BDE 47 (pg/ml)	34.9±9.7	28.9±6.3	39.5±16.5	0.061
BDE 153 (pg/ml)	11.1±2.5	10.3±0.6	11.8±4.4	<0.001
Oxychlordane (pg/mg of lipids)	12.6±0.2	13.9±0.3	11.5±0.2	<0.001
Trans-nonachlor (pg/mg of lipids)	32.9±0.5	39.4±0.9	27.9±0.6	<0.001
PCB 153 (pg/mg of lipids)	319±4	367±8	282±5	<0.001
p,p'-DDE (pg/mg of lipids)	598±15	602±20	596±21	0.303
BDE 47 (pg/mg of lipids)	10.1±2.8	8.1±1.7	11.6±4.7	0.065
BDE 153 (pg/mg of lipids)	3.2±0.3	2.9±0.1	3.4±1.3	<0.001

Data are presented as means±SEM.

° Clinical data refer to the last visit, and POPs levels refer to the baseline visit. Telomere length measurements were available in 1077 subjects, serum lipids in 1080, POPs in 1076, and body composition data by bioimpedance in 1059 subjects.

### Relationship between POPs and telomere outcome/attrition rates in men and women

In men and women, percentile categories were calculated according to the distribution of serum POP levels separately for men and women. Men did not show a clear exposure-response pattern (Fig. 2A). Consistently, glycaemia did not show a significant trend and C-reactive protein showed a U-shaped relationship within the cumulative POPs score categories (Fig. 3B). In women telomere length after 10 years showed a significant reduction across quartile categories of oxychlordane and trans-nonachlor exposure, and a trend across PCB 153 categories (Fig. 2B). Consistently, plasma glucose levels were significantly higher in the highest quartile of the cumulative POPs score and progressively elevated in proportion to, and C-reactive protein showed the same trend (Fig. 3C).

## DISCUSSION

Millions of thousands tons of POPs have been released in the global environment [22, 32]. Peaks were reached progressively after World War II, and restrictions for chlorinated pesticides and PCB oil were enforced from the late seventies to the late eighties. However, due to the persistence of these compounds in the environment and in the human body, their health-related consequences will continue to be seen over many decades to come, and will in the worst case have impact on future generations. POPs have been linked to adverse health outcomes such as cancer, nervous system damage, reproductive disorders, type 2 diabetes, and disruption of the immune system [28]. There is a need to further characterize POPs related outcomes, identify risky exposure-levels and early biomarkers in order to intervene prior to the onset of related diseases (U.S. EPA. 2004a. Pesticides: Regulating pesticides-persistent organic pollutants (POPs). (www.epa.gov/pesticides/regulating/index.htm). Our study population, born between 1934 and 1944 was exposed to the entire period of major emissions, and subsequently to the considerable bioaccumulation and biomagnification occurring in foods, primarily in edible sea-products from Artic, especially the Baltic Sea regions [11, 12, 14, 16, 33]. We focused on the predictive value of POPs on telomere attrition, because the latter is a validated marker of ageing and anticipant of age-related non-communicable diseases [3-9, 34, 35]. To our knowledge, this is the first study to document that serum POPs levels represent a strong predictor of telomere shortening and telomere length in a general population of elderly people. Our findings are in agreement with the available, though still limited, literature evaluating exposure to other toxic environmental chemicals in relation to telomere length [10]. There is one study addressing the correlation between POPs and telomere length in a younger Korean population [36], showing a positive association at low POP levels, and a negative association at high POP levels. However, the study was cross-sectional and small (n=80), and only healthy middle-aged individuals were included, making it difficult to generalize results and extrapolate concepts that are of relevance to the ageing process. Our prospective study was longitudinal, and included more than 1000 randomly selected subjects, truly reflecting the general population through an ageing period of 10 years. Our data show weak cross-sectional correlations between serum POP levels and telomere length at baseline, which may explain the ambiguous finding in the Korean study [36]. Instead, relationships linking serum oxychlordane, trans-nonachlor and PBC153 to telomere length were particularly strong after 10 years of POP measurement, consistent with the long half-lives of these pollutants in humans. Our findings have several important clinical implications. First, they suggest that the detrimental action of POPs on telomere integrity is progressive and delayed. In other words, there seems to be an effective time-window for predicting and preventing the proportion of DNA damage that would be caused by POP exposure. Second, our results indicate that not all POPs may be equally relevant to the long-term modulation of telomere function, since BDEs and p, p’-DDE did not associate with telomere shortening and length at 10 years. In the former case, this may be due to the more rapid elimination of BDE compounds from the human body (half-life of 10-20 days) compared to the other POPs (up to decades) [37]. In fact, association between BDE values and telomere length showed a negative trend at baseline but not in the longer term in the present study, and they were weakly related to the levels of other POPs, as previously reported [28]. The absence of a correlation between serum p, p’-DDE levels and telomere attrition in spite of a long half-life is consistent with studies on DNA integrity in human sperm [38, 39], showing that PCB 153, but not p,p’-DDE might enhance DNA fragmentation. The authors suggested that may be due to PCB 153 metabolites, leading to oxidative DNA damage. A third, clinically important consideration is that the concentrations of POPs observed in our cohort are similar to, or not far from those found in surveys conducted in other European countries and in the US (U.S. Environmental Protection Agency, Blood Persistent Organic Pollutants Level. http://cfpub.epa.gov/roe/indicator.cfm?i=65), including younger people and teenagers [16-20]. Therefore, our findings may extend beyond the boundaries of our cohort, suggesting that the levels of POPs occurring in contemporary younger people may accelerate their DNA ageing from early life stages, leaving them with a longer life-time to experience the consequences of a negative interaction between toxicants and telomeres. This is aggravated by claims of the World Health Organization (WHO, Dioxins and their effects on human health. Fact sheet N°225, (www.who.int/mediacentre/factsheets/fs225/en/) and other expert sources [20], indicating that it is not possible to estimate the degree of human exposure solely based on diet or emission data and region of inhabitance, and that the direct assessment of POPs in serum requires sophisticated and expensive methods that are available only in selected and accredited laboratories in the world. The current evidence of an unequal contribution of different POPs to telomere shortening suggests that a selection of relevant POPs based on the outcome target may be a cost-effective strategy for broader screening.

A noteworthy result of the present study was that there were marked sex-related differences in the relationship linking telomere attrition and POPs levels. In agreement with previous findings [20, 28] women showed 20-40% lower circulating concentrations of oxychlordane, trans-nonachlor and PBC 153 than men. POPs are lipophilic compounds stored primarily in body fat. Women compared to men had a 27% larger mass and 50% greater percentage of adipose tissue. We observed a strong negative correlation between POPs levels and the relative amount of body fat, which partly explained the sex-related differences in circulating POPs concentrations. Adipose tissue may serve as a protective sink for these toxicants, but is also a chronic internal source of POPs [40, 41]. Interestingly, rapid weight loss in humans and animals is accompanied by an increase in serum POPs levels [41]. Our subjects were not obese, but men underwent some degree of weight loss during the study period whereas women did not (data not shown). In men, circulating oxychlordane, trans-nonachlor and PBC 153 were higher than in women both when expressed as wet-weight or lipid-standardized (Table 1), and not association was observed between serum trans-nonachlor or PBC153 levels and telomere shortening, suggesting that POPs might affect telomere length in men with a threshold effect. In women, telomeres were longer, and their relationship with POPs was more gradual. Across quartiles there was a significant trend, i.e. a progressive decline in telomere length in proportion to the level of exposure, and in group comparison the quartile with the highest exposure had significantly shorter telomeres compared to the quartile with the lowest exposure. These data suggest that the buffering role of adipose tissue may explain the differential exposure-response pattern observed in men and women, affecting their telomere vulnerability to POPs.

**Table 2:** Relationships between serum POPs levels and telomere shortening (n=1047) or telomere length at 10 years (n=1071)

Serum POPs levels	Telomere shortening (Δ in 10 years)		Telomere length (at 10 years)		Adjustment (multivariate)
	r	*p*	r	*p*	
**Serum pg/ml (wet weight)**					
Cumulative POPs levels^°^	-0.069	**0.027**	-0.093	**0.002**	**-**
	-0.050	0.109	-0.082	**0.008**	Lipids
	-0.052	0.091	-0.090	**0.003**	Lipids + Δ Age
	-0.033	0.299	-0.070	**0.024**	Lipids + Body Fat %
	-0.035	0.267	-0.074	**0.017**	Lipids + Δ Age + Body Fat %
	-0.031	0.326	-0.061	**0.052**	Lipids+ Δ Age + Body Fat % + Diseases
					Lipids + Δ Age + Diseases
	-0.051	0.100	-0.080	**0.009**	
Oxychlordane	-0.063	**0.041**	-0.078	**0.011**	**-**
	-0.056	0.072	-0.086	**0.005**	Lipids
	-0.057	0.068	-0.098	**0.004**	Lipids + Δ Age
	-0.043	0.178	-0.074	**0.017**	Lipids + Body Fat %
	-0.043	0.176	-0.075	**0.016**	Lipids+ Δ Age + Body Fat %
	-0.041	0.201	-0.062	**0.047**	Lipids+ Δ Age + Body Fat % + Diseases
					Lipids + Δ Age + Diseases
	-0.056	0.073	-0.079	**0.011**	
Trans-nonachlor	-0.083	**0.007**	-0.090	**0.003**	**-**
	-0.070	**0.024**	-0.095	**0.002**	Lipids
	-0.072	**0.021**	-0.098	**0.001**	Lipids + Δ Age
	-0.055	0.081	-0.080	**0.010**	Lipids + Body Fat %
	-0.056	0.078	-0.082	**0.008**	Lipids+ Δ Age + Body Fat %
	-0.053	0.094	-0.068	**0.030**	Lipids+ Δ Age + Body Fat % + Diseases
					Lipids + Δ Age + Diseases
	-0.071	**0.023**	-0.088	**0.004**	
PCB 153	-0.064	**0.038**	-0.090	**0.003**	**-**
	-0.046	0.140	-0.081	**0.008**	Lipids
	-0.049	0.117	-0.086	**0.005**	Lipids + Δ Age
	-0.029	0.357	-0.067	**0.032**	Lipids + Body Fat %
	-0.032	0.318	-0.071	**0.023**	Lipids+ Δ Age + Body Fat %
	-0.038	0.384	-0.058	0.065	Lipids+ Δ Age + Body Fat % + Diseases
					Lipids + Δ Age + Diseases
	-0.047	0.128	-0.077	**0.012**	
p,p'-DDE	0.009	0.766	-0.030	0.325	
BDE 47	0.027	0.718	-0.013	0.681	
BDE 153	-0.019	0.807	-0.016	0.608	
**Quartiles**					
Cumulative POPs	-0.076	**0.013**	0.088	**0.004**	**-**
levels^°^	-0.064	**0.038**	-0.093	**0.003**	Lipids
	-0.066	**0.032**	-0.096	**0.002**	Lipids + Δ Age
	-0.049	0.120	-0.076	**0.016**	Lipids + Body Fat %
	-0.050	0.112	-0.078	**0.014**	Lipids+ Δ Age + Body Fat %
	-0.047	0.134	-0.066	**0.038**	Lipids+ Δ Age + Body Fat % + Diseases
					Lipids + Δ Age + Diseases
	-0.066	**0.034**	-0.089	**0.004**	
Oxychlordane	-0.063	**0.040**	-0.090	**0.003**	**-**
	-0.051	0.097	-0.097	**0.002**	Lipids
	-0.052	0.093	-0.098	**0.001**	Lipids + Δ Age
	-0.040	0.201	-0.087	**0.005**	Lipids + Body Fat %
	-0.040	0.202	-0.087	**0.005**	Lipids+ Δ Age + Body Fat %
	-0.038	0.228	-0.076	**0.015**	Lipids+ Δ Age + Body Fat % + Diseases
					Lipids + Δ Age + Diseases
	-0.051	0.098	**-0.091**	**0.003**	
Trans-nonachlor	-0.084	**0.007**	0.092	**0.003**	**-**
	-0.079	**0.011**	-0.106	**0.001**	Lipids
	-0.080	**0.009**	-0.108	**<0.001**	Lipids + Δ Age
	-0.065	**0.041**	-0.093	**0.003**	Lipids + Body Fat %
	-0.065	**0.038**	-0.095	**0.002**	Lipids+ Δ Age + Body Fat %
	-0.063	**0.047**	-0.083	**0.008**	Lipids+ Δ Age + Body Fat % + Diseases
					Lipids + Δ Age + Diseases
	-0.080	**0.010**	-0.101	**0.001**	
PCB 153	-0.066	**0.034**	-0.100	**0.001**	**-**
	-0.050	0.106	-0.097	**0.001**	Lipids
	-0.063	0.087	-0.103	**0.001**	Lipids + Δ Age
	-0.032	0.311	-0.084	**0.007**	Lipids + Body Fat %
	-0.035	0.273	-0.088	**0.005**	Lipids+ Δ Age + Body Fat %
	-0.032	0.297	-0.078	**0.012**	Lipids+ Δ Age + Body Fat % + Diseases
					Lipids + Δ Age + Diseases
	-0.054	0.085	-0.097	**0.002**	
p,p'-DDE	0.001	0.981	-0.037	0.224	
BDE 47	0.026	0.397	-0.014	0.638	
BDE 153	-0.011	0.727	0.009	0.769	

° Cumulative refers to the sum of the 3 POPs showing significant relationships with telomere length (oxychlordane, trans-nonachlor, PBC 153); multivariate regression analyses (right column) were performed only when a significant relationship was observed in univariate analyses; lipids refer to the sum of total cholesterol and triglycerides serum concentrations at the time of POPs measurements, Δ Age refers to the interval occurring between the baseline and follow-up visit; fat percent refers to the assessment of body composition done at the time of POPs measurements; significant p values are in bold font.

In order to examine if adiposity could entirely account for the relationship between serum POPs and telomere length at the whole cohort level, we performed multivariate regression analysis, and found that POPs remained strong significant predictors of telomere attrition, independent of adiposity and age. Therefore, a greater body fat percentage may partly account for the observed sex-related differences, but does not explain the apparent detrimental effects of POPs on telomeres. One recognized cause of telomere damage is inflammation [40]. Interestingly, inflammation is also induced by POPs [41, 42], and is one main proposed mechanism to explain their association with chronic diseases [42, 43]. In our dataset, baseline levels of C-reactive protein mirrored the distribution of telomere length and shortening across percentile categories in the whole study population and in women. In men, CRP levels did not show a linear relationship with POPs exposure, while in women a positive trend between CRP levels and POP exposure was found. Another mechanism linking POPs and chronic diseases is endocrine disruption, involving reproductive and metabolic hormones. We have previously reported that serum POPs concentrations were related to the likelihood of having type 2 diabetes in a larger sample of the present cohort [28]. Longitudinal studies have shown that telomere length predicts the development of type 2 diabetes [31, 34, 35]. In our study, plasma glucose was already elevated in proportion to POPs levels at baseline, i.e. prior to telomere shortening. Altogether, the previous and current findings support the involvement of inflammation and hyperglycemia in the association linking pollutants and telomere attrition.

The main strength of the present study is the double measurement of telomere length at two time points in a cohort of more than 1000 subjects. The study population is ethnically homogenous, which is both a strength and a weakness. Because of this the results might not be generalized to other populations. Since many years have passed, different method have been used for telomere length measurement and we cannot exclude that small technical differences might have an effect on absolute T/S values. However, the error, if any, would be systematic, and have limited influence on group comparisons and associations involving relative LTL change rates. In fact, we observed a correlation between LTL at the two time-points (p<0.001). We acknowledge that the baseline measurement was affected by a greater technical variability, which might have obscured some significance in the statistical analysis.

In conclusion, our study provides solid evidence that the exposure to POPs, and in particular oxychlordane, trans-nonachlor and PCB 153 predicts telomere attrition. Since shorter telomeres affect gene repair in all body organs, this mechanism may explain the variety of diseases that have been associated to the exposure to these toxicants in humans. Noteworthy, values of POPs occurring in contemporary younger people are in the same order of magnitude as the ones observed here, and may potentially contribute to an accelerated ageing, starting from earlier life stages. Finally, the observation that not all POPs were similarly toxic to telomeres offers a strategy to reduce the high costs associated to POPs assays, by selecting screening panels of POPs that are relevant to the specific outcome of interest.
